# Effect of the COVID-19 pandemic restrictions on outcomes of HIV care among adults in Uganda

**DOI:** 10.1097/MD.0000000000030282

**Published:** 2022-09-09

**Authors:** Jonathan Izudi, Agnes N. Kiragga, Philip Kalyesubula, Stephen Okoboi, Barbara Castelnuovo

**Affiliations:** a Infectious Diseases Institute (IDI), Makerere University College of Health Sciences, Kampala, Uganda; b Department of Community Health, Faculty of Medicine, Mbarara University of Science and Technology, Mbarara, Uganda; c Data Science and Evaluations (DSE) Unit, African Population and Health Research Center (APHRC), Nairobi, Kenya.

**Keywords:** COVID-19 lockdown, COVID-19 restrictions, impact of COVID-19, Uganda

## Abstract

Uganda enforced several stringent restrictions such as night-time curfews, travel bans, school closure, and physical and social distancing among others that constituted a national lockdown to prevent the spread of the Coronavirus disease 2019 (COVID-19). These restrictions disrupted the delivery of health services but the impact on outcomes of human immunodeficiency virus (HIV) care has not been rigorously studied. We evaluated the effect of the COVID-19 pandemic restrictions on outcomes of HIV care among people living with HIV (PLHIV) aged ≥15 years in Kampala, Uganda. We designed a nonrandomized, quasi-experimental study using observational data retrieved from six large HIV clinics and used the data to construct two cohorts: a comparison cohort nonexposed to the restrictions and an exposed cohort that experienced the restrictions. The comparison cohort consisted of PLHIV commenced on anti-retroviral therapy (ART) between March 1, 2018, and February 28, 2019, followed for ≥1 year with outcomes assessed in March 2020, just before the restrictions were imposed. The exposed cohort comprised of PLHIV started on ART between March 1, 2019, and February 28, 2020, followed for ≥1 year with outcomes assessed in June 2021. The primary outcomes are retention, viral load testing, viral load suppression, and mortality. We employed inverse probability of treatment weighting using propensity score (IPTW-PS) to achieve comparability between the two cohorts on selected covariates. We estimated the effect of the restriction on the outcomes using logistic regression analysis weighted by propensity scores (PS), reported as odds ratio (OR) and 95% confidence interval (CI). We analyzed data for nine, 952 participants, with 5094 (51.2%) in the exposed group. The overall mean age was 32.7 ± 8.8 years. In the exposed group relative to the comparison group, viral load testing (OR, 1.68; 95% CI, 1.59–1.78) and viral load suppression (OR, 1.34; 95% CI, 1.110–1.63) increased while retention (OR, 0.76; 95% CI, 0.70–0.81) and mortality (OR, 0.75; 95% CI, 0.64–0.88) reduced. Among PLHIV in Kampala, Uganda, viral load testing and suppression improved while retention and mortality reduced during the COVID-19 pandemic restrictions due to new approaches to ART delivery and the scale-up of existing ART delivery models.

## 1. Introduction

On March 21, 2020, three days before confirming the first case of coronavirus disease of 2019 (COVID-19) from an international traveler, Uganda enforced several stringent restrictions like night-time curfews, travel bans, school closure, and physical and social distancing constituting a national lockdown.^[1]^ The restrictions were meant to limit the spread of severe acute respiratory syndrome coronavirus 2. The national lockdown stretched from March 18, 2020, to June 4, 2020, causing severe social and economic disruptions nationwide. Within the health sector, the main disruptions include re-deployment of the existing health workforce to respond to the pandemic, difficult access to health facilities due to the restrictions (suspension of public transport, curfew at 7 pm, restriction on the carrying capacity of private vehicles) and a shift in healthcare priority to pandemic control and prevention.^[2]^ These disruptions negatively impacted maternal, neonatal, child, sexual, and reproductive health services in the earlier phases (Mar-Apr 2020) of the pandemic response.^[3,4]^

Uganda has an estimated 1.4 million people living with human immunodeficiency virus (PLHIV)^[5]^ and of this, 88% know their HIV status, 87% of those with known HIV status are on anti-retroviral therapy (ART)^[5]^ and 90% of those on ART have suppressed viral load.^[6]^ Disruptions to HIV service delivery by the COVID-19 pandemic restrictions are likely to reverse these gains. Engagement and continuity of care are important in human immunodeficiency virus (HIV) care for individual patients’ outcomes and public health prevention. Consequently, all services along the HIV cascade (HIV testing, linkage to care, uptake of ART, and treatment monitoring) should be accessible.^[7]^ Anecdotal observations suggest the disruptions have led to PLHIV experiencing longer waiting times at health facilities, not adhering to clinic visits, and receiving inadequate care from healthcare providers. Besides, PLHIV experienced compromised access to ART including ART adherence support and this might negatively impact retention and viral load suppression.

A study conducted across 65 primary health care facilities in South Africa to measure the impact of the COVID-19 restrictions on HIV care clinics found that such restrictions decreased HIV testing and ART initiations but had no remarkable impact on the number of ART collection visits.^[8]^ The easing of the restrictions led to improvements in HIV testing and ART initiations towards pre-restriction levels.^[8]^ In Uganda, a prediction model estimates that the direct impact of the pandemic might relatively be low due to a younger population but the risk of negative impact on non-COVID-19 diseases (tuberculosis, malaria, and HIV) might probably be more pronounced due to prolonged restrictions.^[9]^ Reports suggest that the most important effects of the pandemic on population health will be more indirect than direct.^[2,10]^ For example, through interruptions to the delivery of HIV, tuberculosis, and malaria services.^[2,10,11]^ Currently, there is limited information from rigorously designed studies regarding the impact of the COVID-19 pandemic restrictions on HIV care outcomes in Uganda. We evaluated the effect of the COVID-19 pandemic restrictions on retention, viral load access, viral load suppression, and mortality among PLHIV aged ≥15 years in Kampala city, Uganda.

## 2. Methods

### 2.1. Study design

We designed a nonrandomized, quasi-experimental study using observational data since it was not feasible to use a randomized control trial to evaluate the impact of the COVID-19 pandemic restrictions on HIV care. Observational data have limitations in establishing cause-effect (causality) due to selection bias^[12]^ and confounding.^[13]^ Therefore, to approximate an randomized control trial, we utilized inverse probability of treatment weighting using propensity scores (IPTW-PS) to create a comparison (nonexposed) group which was similar to the exposed group on all measured covariates except for the exposure. The propensity score (PS) is the probability of being in the exposed group conditional on the observed participant’s characteristics.^[12]^ IPTW-PS is a statistical approach that removes selection bias and confounding by weighting the exposed and nonexposed groups using PS. Since we did not use true randomization to achieve comparability, this is a quasi-experimental study.^[14]^

### 2.2. Ethical issues

The Infectious Diseases Institute Research Ethics Committee (reference number 013/2021) and Uganda National Council for Science and Technology (reference number HS709ES) approved the study. The committee granted a waiver of informed consent since secondary data were retrieved and analyzed, and because it was logistically infeasible to track the participants for informed consent. The Directorate of Public and Environmental Health of Kampala Capital City Authority (reference number DPHE/KCCA/1301) provide administrative approval.

### 2.3. Study setting and participants

We retrieved electronic health records for the period March 1, 2018, to February 28, 2020, from six health facilities within the Kampala Capital City Authority namely Kisenyi Health Center IV (HC IV), Kawaala HC III, Kisugu HC III, Kitebi HC III, Komamboga HC III, and Kiswa HC III. HC IV is a County level health facility designated to serve a 100,000 population. It provides basic preventive, curative, and rehabilitative care including referral services for life-saving medical, surgical and obstetrical emergency care such as blood transfusion, cesarean section, and other medical and surgical emergency interventions.^[15]^ HC III is a Sub-country level health facility that serves 20,000 people and provides basic preventive, promotive, and curative care including technical support to lower-level facilities. The sites have provisions for laboratory services, maternity care, and first-level referral cover for the Sub-county.^[15]^ Each of the study sites has an HIV clinic managed by an HIV/ART focal person, namely a medical doctor, clinical officer, or nursing officer. The provision of HIV care follows the national HIV treatment guidelines.^[5]^ HIV data are captured at every clinic visit using a paper-based HIV/ART card and entered into an electronic open medical records system.

We included data for PLHIV aged ≥15 years enrolled in care between March 01, 2018, and February 28, 2020. We excluded participants transferred to other health facilities since it was logistically inefficient to trace them. We used the data to create two groups, a comparison, and an exposed group. The comparison group consisted of PLHIV who were not exposed to the COVID-19 pandemic restrictions.

Here, we considered those initiated on ART between March 1, 2018, and February 28, 2019, followed for at least 1 year with outcomes assessed in March 2020 just before the COVID-19 pandemic restrictions. The exposed group consisted of PLHIV who experienced the COVID-19 pandemic restrictions. We considered those started on ART between March 1, 2019, and February 28, 2020, followed for at least 1 year, with outcomes assessed in June 2021.

### 2.4. Study outcomes

• Viral load testing: Receiving a viral load test while in care (excluding viral load test at 6 months).^[16]^
• Viral load suppression: A viral load <1000 copies/mL among those who had received viral load testing.^[16]^
• Retention: Participants with a visit to the HIV clinic at least once within the last four weeks from the date of the last scheduled visit.^[17]^
• All cause-mortality: Participants documented or reported to have died.^[16]^

### 2.5. Covariates

We selected nine baseline covariates associated with the study outcomes to generate PS. This approach reduces selection bias and improves the precision of effect estimates.^[18]^ The covariates were district of residence (Kampala, Wakiso, and others), study sites (Kisenyi HCIV, Kawaala HCIII, Kisugu HCIII, Kitebi HCIII, Komamboga HCIII, and Kiswa HCIII), level of health facility (HC II versus HC IV), sex (male versus female), age measured in absolute years and later categorized into 15 to 24, 25 to 34, 35 to 44, and ≥55 years, and point of entry into HIV care. The points of entry included the in-patient department, Out-patient department, Outreach, Prevention of Mother to Child Transmission of HIV Clinic, safe male circumcision Clinic, Sexually Transmitted Infections Clinic, Tuberculosis Clinic, and the Young Child Clinic. The other entry points were index client contact testing like assisted partner notification services, social network testing services, client-initiated testing and counseling, home-based HIV counseling and testing, and index client testing). Other covariates included baseline ART regimen (TDF-3TC-EFV, TDF-3TC-DTG, and other first-line ART regimens) and baseline weight in kilograms.

### 2.6. Statistical methods

We hypothesized that viral load testing, viral load suppression, and retention rates would decrease during the COVID-19 pandemic restrictions while mortality would increase. We performed the analysis in R version 4.0.2 (2020-06-22).^[19]^ We summarized baseline categorical data using frequencies and percentages and numerical data using means with standard deviation. We performed IPTW-PS to create two identical groups on observed covariates with the “*PSW package*.”^[20]^ We generated PS in a logistic regression model by regressing the exposure on the covariates. The PS is the probability of being in the exposed group conditional on the observed participant’s characteristics.^[12]^ We assessed balance in PS across the groups using a back-to-back histogram, with distributional similarity in PS and weights interpreted as suggestive of good covariate balance. We used the estimated PS to weight the groups, with the exposed group weighted using the inverse of PS (1/PS) and the comparison group weighted using the inverse of one minus the PS (1/(1 − PS)). This created a pseudo-population with balanced covariates. We assessed covariate balance using standardized mean difference (SMD) and those with SMD ≤ 0.1 were considered balanced.^[21–23]^ After covariate balance, we estimated the exposure-outcome effect using PS weighted logistic regression, reported as odds ratio (OR) and 95% confidence interval (CI).

### 2.7. Additional analysis

We used multiple imputations with chained equations to impute variables with more than 10% missing data and assumed that data were missing at random. We assessed the sufficiency of the PS model specification through a model specification test, with the null hypothesis that the model was correctly specified.^[21]^ We assessed the robustness of the estimates to unmeasured confounders using sensitivity analysis.^[22]^ Here, a distant Gamma value for a shift from statistically significant to statistically insignificant value (or vice-versa) in the odds of the upper or lower bounds was considered indicative of robust findings.^[22]^ We reported the findings following guidelines for PS analysis.^[24]^ Our findings are reported following the improving the reporting quality of nonrandomized evaluations of behavioral and public health interventions: The TREND statement.^[25]^

## 3. Results

### 3.1. Study profile

**Figure 1.**
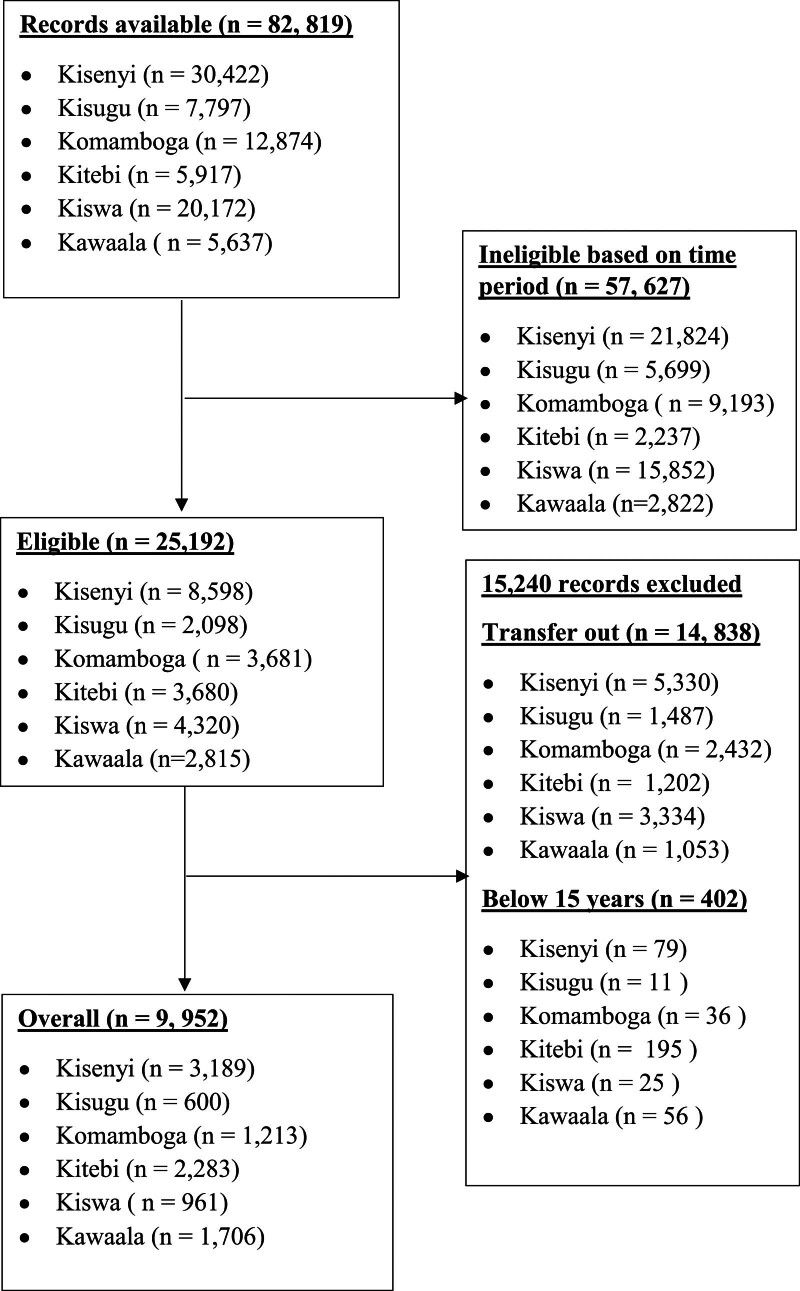
Study flowchart for the effect of COVID-19 restrictions on HIV care in Kampala, Uganda. COVID-19 = coronavirus disease 2019.

We retrieved 82,819 records and excluded 57,627 of them because they fell outside the period of March 1, 2018, and February 28, 2020. Of the remaining records, we excluded 14,838 records for participants who were transferred to other health facilities and another 402 records for participants <15 years. Therefore, we analyzed data for 9952 participants: 4858 (48.8%) in the comparison group versus 5094 (51.2%) in the exposed group (Fig. 1).

### 3.2. Characteristics of participants

The overall mean age was 32.7 ± 8.8 years and participants in the exposed group were on average slightly younger than those in the comparison group: 32.0 ± 7.0 versus 33.4 ± 8.6, respectively (*P* < .001). The majority of the participants were residents of Kampala district (6605, 66.4%), from Kisenyi HC IV (3189, 32.0%), had enrolled into HIV care through the Outpatient Department (5201, 52.3%), females (6596, 66.3%), aged 25 to 34 years (49.3%), and on TDF/3TC/DTG (55.4%). The two groups were systematically different regarding the study sites, level of health facility, entry point into HIV care, age, and baseline ART regimen, all with *P* < .001.

### 3.3. Results of study outcomes between the exposed and comparison groups

Table 1 shows that fewer participants (33.1%) in the comparison group had viral load testing compared to the exposed group (44.2%) (*P* < .001). Of those tested for viral load, fewer patients in the exposed group (66.7%) had viral suppression as compared to the comparison group (94.0%). We found that 1901 (19.1%) participants were retained in care, with fewer participants retained in care in the exposed group (17.3%) than in the comparison group (21%) (*P* < .001). More participants in the comparison group (3.9%) had died compared to the exposed group (2.7%) (*P* = .001).

**Table 1:** Characteristics of participants.

Variables	Level	Overall (n = 9952)	Comparison group (n = 4858)	Exposed group (n = 5094)	*P* value
District of residence	Kampala	6605 (66.4)	3228 (66.4)	3377 (66.3)	.232
	Wakiso	2869 (28.8)	1380 (28.4)	1489 (29.2)	
	Others	478 (4.8)	250 (5.1)	228 (4.5)	
Study site	Kawaala HC III	1706 (17.1)	920 (18.9)	786 (15.4)	<.001
	Kisenyi HC IV	3189 (32.0)	1681 (34.6)	1508 (29.6)	
	Kisugu HC III	600 (6.0)	310 (6.4)	290 (5.7)	
	Kiswa HC III	961 (9.7)	488 (10.0)	473 (9.3)	
	Kitebi HC III	2283 (22.9)	808 (16.6)	1475 (29.0)	
	Komamboga HC III	1213 (12.2)	651 (13.4)	562 (11.0)	
Level of health facility	HC III	6763 (68.0)	3177 (65.4)	3586 (70.4)	<.001
	HC IV	3189 (32.0)	1681 (34.6)	1508 (29.6)	
Point of entry point into HIV care	In-patient department	11 (0.1)	4 (0.1)	7 (0.1)	<.001
	Outpatient department	5201 (52.3)	2791 (57.5)	2410 (47.3)	
	Outreach	182 (1.8)	95 (2.0)	87 (1.7)	
	PMTCT	1711 (17.2)	842 (17.3)	869 (17.1)	
	SMC clinic	16 (0.2)	13 (0.3)	3 (0.1)	
	STI clinic	3 (0.0)	0 (0.0)	3 (0.1)	
	TB clinic	297 (3.0)	147 (3.0)	150 (2.9)	
	Young child clinic	24 (0.2)	12 (0.2)	12 (0.2)	
	Others*	2507 (25.2)	954 (19.6)	1553 (30.5)	
Sex	Female	6596 (66.3)	3201 (65.9)	3395 (66.6)	.4328
	Male	3356 (33.7)	1657 (34.1)	1699 (33.4)	
Age categories (yr)	15–24	1543 (15.5)	594 (12.2)	949 (18.6)	<.001
	25–34	4903 (49.3)	2394 (49.3)	2509 (49.3)	
	35–44	2473 (24.8)	1340 (27.6)	1133 (22.2)	
	45 and beyond	1033 (10.4)	530 (10.9)	503 (9.9)	
	mean (SD)	32.7 (8.8)	33.4 (8.6)	32.0 (7.0)	<.001
Baseline ART regimen	TDF-3TC-EFV	5514 (55.4)	3441 (70.8)	2073 (40.7)	<.001
	TDF-3TC-DTG	1921 (19.3)	493 (10.1)	1428 (28.0)	
	Other first-line regimens	2517 (25.3)	924 (19.0)	1593 (31.3)	
Baseline weight (kg)	Mean (SD)	63.5 (17.8)	63.70 (18.3)	63.3 (17.2)	.235
Viral load testing	No	6090 (61.2)	3250 (66.9)	2840 (55.8)	<.001
	Yes	3862 (38.8)	1608 (33.1)	2254 (44.2)	
Viral load suppressed† (n = 3862)	No	204 (10.6)	97 (6.0)	107 (33.3)	<.001
	Yes	3658 (89.4)	1511 (94.0)	2147 (66.7)	
Retention in care	No	8051 (80.9)	3837 (79.0)	4214 (82.7)	<.001
	Yes	1901 (19.1)	1021 (21.0)	880 (17.3)	
Mortality	No	9626 (96.7)	4669 (96.1)	4957 (97.3)	.001
	Yes	326 (3.3)	189 (3.9)	137 (2.7)	

HC = health center, PMTCT = Prevention of Mother to Child Transmission of HIV, SD = standard deviation, SMC = safe male circumcision, STI = sexually transmitted infections, TB = tuberculosis, TDF-3TC-DTG = Tenofovir, Lamivudine, and Dolutegravir, TDF-3TC-EFV = Tenofovir, Lamivudine, and Efavirenze.

* Index client contact testing such as assisted partner notification services, social network testing services, client-initiated testing and counselling, home-based HIV counselling and testing, and index client testing or index case HIV testing.

† Data are for those tested for viral load.

### 3.4. Balance diagnostics

#### 3.4..1. Distribution of PS.

**Figure 2.**
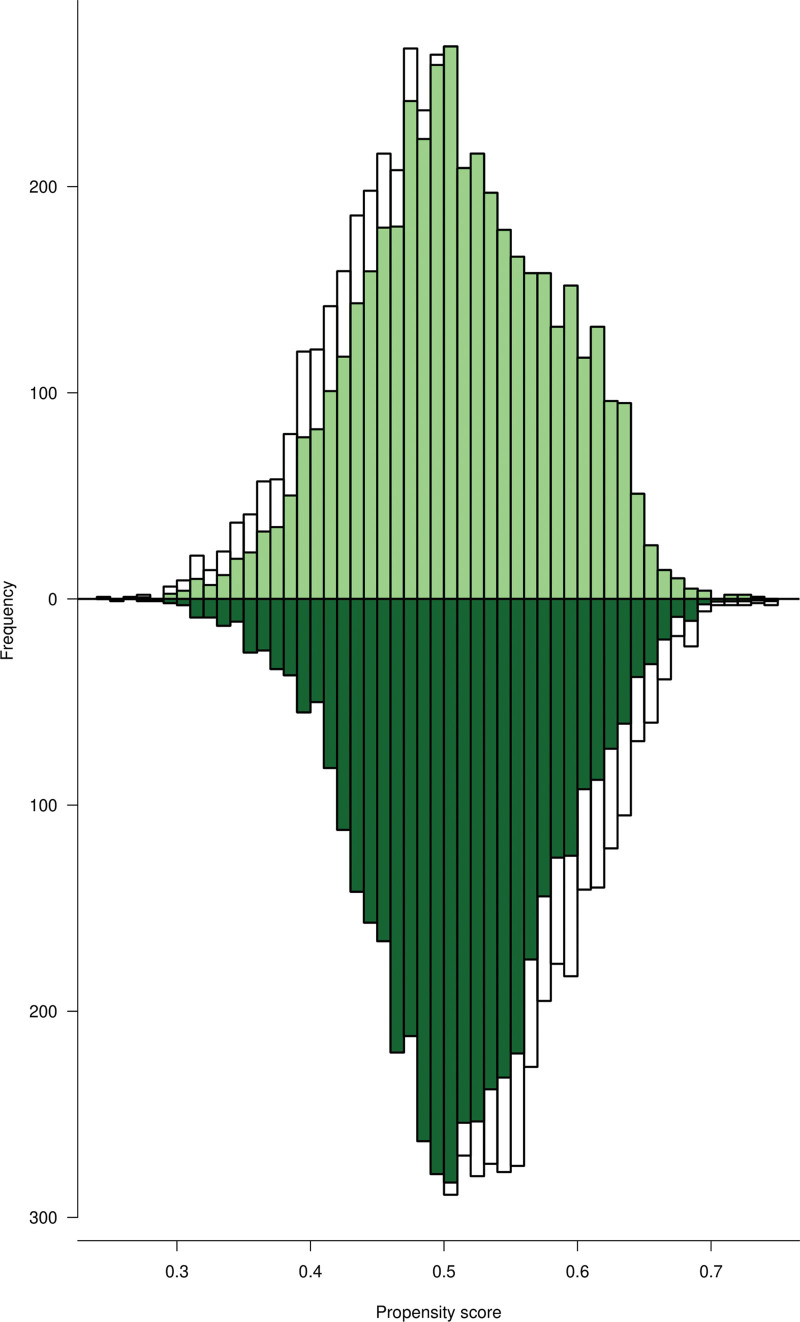
Mirror-histogram showing the distribution of PS between the comparison and exposed groups. PS = propensity scores.

Figure 2 shows the distribution of PS in a mirror-histogram for both the comparison and exposed groups. The upper section of the histogram (light green) shows the distribution of PS in the comparison group while the lower section (dark green) depicts that in the exposed group. The PS ranged from 0.24 to 0.75.^[26]^ We observed distributional similarity in PS across the groups after weighting.

#### 3.4..2. SMD before and after IPTW-PS.

Table 2 shows additional balance diagnostics using SMD before and after IPTW-PS. Systematic differences (covariate imbalance) between the two groups were observed before IPTW-PS concerning the study sites (SMD = 0.168), level of health facility (SMD = 0.107), and age categories (SMD = 0.162) and age in absolute years (SMD = 0.178). These systematic differences were removed after IPTW-PS, with all the covariates having an SMD < 0.1.

**Table 2:** Balance diagnostics using SMD before and after IPTW-PS.

Characteristics	Before IPTW-PS					After IPTW-PS				
	Exposed group		Comparison group			Exposed group		Comparison group		
	Mean	SD	Mean	SD	SMD	Mean	SD	Mean	SD	SMD
District of residence	1.63	0.90	1.62	0.90	0.011	1.62	0.91	1.62	0.90	0.003
Study site	1.06	0.61	0.96	0.62	0.168	1.01	0.61	1.01	0.62	0.002
Level of health facility	1.30	0.46	1.35	0.48	−0.107	1.32	0.47	1.32	0.47	−0.005
Age categories (yr)	3.23	0.87	3.37	0.83	−0.162	3.30	0.87	3.30	0.81	<0.001
Age (yr)	3.43	0.26	3.48	0.25	−0.178	3.46	0.26	3.45	0.24	0.001
Sex	1.33	0.47	1.34	0.47	−0.016	1.33	0.47	1.33	0.47	−0.002
Point of entry into HIV care	3.05	1.42	2.96	1.47	0.058	3.01	1.40	3.01	1.48	<0.001
Baseline regimen	1.97	0.77	1.91	0.53	0.085	1.94	0.77	1.94	0.52	0.001
Baseline weight	4.12	0.23	4.12	0.25	−0.014	4.12	0.23	4.12	0.24	−0.003

HIV = human immunodeficiency virus, IPTW-PS = inverse probability of treatment weighting using propensity scores, SD = standard deviation, SMD = standardized mean difference.

#### 3.4..3. Effects of the COVID-19 pandemic restrictions on study outcomes.

Table 3 shows the effect of the COVID-19 pandemic restrictions on the study outcomes. Before IPTW-PS, the data show viral load testing (OR, 1.60; 95% CI, 1.48–1.74) and viral load suppression (OR, 1.29; 95% CI, 0.97–1.71) were more likely in the exposed group than the comparison group but retention (OR, 0.78; 95% CI, 0.71–0.87) and mortality (OR, 0.68; 95% CI, 0.55–0.85) were less likely in the exposed group than the comparison group. After IPTW-PS, the data show that viral load testing (OR, 1.68; 95% CI, 1.59–1.78) and viral load suppression (OR, 1.35; 95% CI, 1.11–1.64) improved in the exposed group than the comparison group, while retention (OR, 0.76; 95% CI, 0.70–0.81) and mortality (OR, 0.75; 95% CI, 0.64–0.88) reduced in the exposed group relative to the comparison group.

**Table 3:** Effects of the COVID-19 pandemic restrictions on HIV care.

Variable	Level	Logistic regression analysis before IPTW-PS	Logistic regression analysis after IPTW-PS
		OR (95% CI)	OR (95% CI)
Access to viral load testing	No	1	1
	Yes	1.60*** (1.48, 1.74)	1.68*** (1.59, 1.78)
Viral load suppression	No	1	1
	Yes	1.29 (0.97, 1.71)	1.35** (1.11, 1.64)
Retention in care	No	1	1
	Yes	0.78*** (0.71, 0.87)	0.76*** (0.70, 0.81)
Mortality	No	1	1
	Yes	0.68** (0.55, 0.85)	0.75*** (0.64, 0.88)

CI = confidence interval, COVID-19 = coronavirus disease 2019, HIV = human immunodeficiency virus, IPTW-PS = inverse probability of treatment weighting using propensity scores, OR = odds ratio.

*P < .05.

** *P* < .01.

*** *P* < .001.

### 3.5. Additional results

#### 3.5..1. Model specification test and sensitivity analysis.

The PS model specification test showed the null hypothesis could not be rejected (*t* test = 13.70, degree of freedom = 9, *P* = .133), suggesting a correct model specification. In the sensitivity analysis, there was a shift from statistically significant to statistically insignificant value in the upper bounds when the gamma value was 1.25. Therefore, the point of statistical insignificance was distant from 1.0, a point of no hidden bias from unmeasured covariates. This implied the results are not sensitive to unmeasured covariates/or hidden bias hence robust.

## 4. Discussion

Our study on the effect of the COVID-19 pandemic restrictions on HIV care outcomes in Kampala, the capital city of Uganda, surprisingly showed that viral load testing and suppression improved, while mortality and retention reduced during the COVID-19 pandemic restrictions. These findings are explained by several factors. First, during the restrictions, the MoH provided swift guidance concerning the continuity of essential health services at the national, district, health facility, and community levels. At the national level, guidelines for continuity of HIV services amidst the restrictions were developed, followed by national dissemination through district-based training. The training raised the confidence of healthcare workers in providing HIV services amidst the restrictions. MoH developed and disseminated 15 guidelines to ensure continuity of health services during the restrictions and six of the guidelines focused on HIV, namely the guidance on continuity of essential health services during the COVID-19 outbreak; guidance on HIV prevention, diagnostic, and treatment activities in the context of COVID-19; guidance on tuberculosis prevention, diagnostic and treatment activities in the context of COVID-19; guidelines on providing mental health and psychosocial care during COVID-19 pandemic response; guidelines on provision of sexual reproductive health, HIV and gender-based violence services in the context of COVID-19; and, guidance on home-based care and continuity of essential health services in the face COVID-19 pandemic.

Second, the Presidential Emergency Plan for AIDS Relief (PEPFAR) guided the continuity of HIV services by providing specific guidance on HIV testing, treatment and prevention, and HIV pre-exposure prophylaxis amongst others.^[27]^ The Uganda PEPFAR data show viral load testing coverage was 71% during the restrictions (January to March 2020) compared to 78% before the restrictions (September to December 2019), suggesting the restrictions had no significant effect on viral load testing. Furthermore, PEPFAR data indicated a 92% viral load suppression during the restrictions (April to June 2020) compared to 91% before the restrictions (October 2019 to March 2020), suggesting little improvement in viral suppression during the restrictions.^[28]^ Our finding that viral load testing and suppression improved is consistent with the Uganda PEPFAR data.

Third, at the district and community levels, several differentiated service delivery models which were approved by the MOH for the distribution of HIV medications were scaled up. Notable examples include the community distribution of drugs where HIV medications are delivered at designated points within the community; drop-in methods where PLHIV picked medications at the nearest health facility; and, the use of motorcycle taxis, and courier services. Elsewhere,^[29]^ PLHIV under the community-based drug distribution model had nearly perfect ART adherence. Besides, health facilities adopted multi-month prescription and dispensing of HIV medications where those with stable viral load are prescribed and dispensed treatment for 4 to 6 months instead of 1 to 2 months. This approach offered protection against loss to follow-up and ART nonadherence for the period the ARVS had been dispensed. A previous study conducted on PLHIV and hypertension in Kampala, Uganda reports access to HIV medicines remained nearly universal during the restrictions, with 49% to 66% of those with missed clinic appointments seeking care at the nearest health facility.^[30]^ Specifically, in our setting, the health facilities adopted a pharmacy-community dispensing approach where mobile ART clinics were conducted for PLHIV with difficult access to health facilities. This led to sustained ART delivery and ART adherence hence viral load suppression. In Western Uganda, a mobile ART pharmacy for PLHIV with difficult access to health facilities led to improved ART adherence and viral load suppression,^[31]^ consistent with our findings. Other innovations included home-based delivery of HIV medications by health workers, expert clients, peers, and health facility linkage facilitators.

This approach was guided by patient locator information obtained at the time of enrolment into HIV care and active tracking of those with missed appointments.

The observed improvements in viral load testing and suppression are consistent with a previous study.^[32]^ The study attributed the improvements to increased frequency of viral load specimen pick-ups at health facilities, the expansion of dried blood spot specimen collections that enabled easy storage and transportation without the need for refrigeration, and mobilization of a network of PLHIV to serve as community volunteers in collecting ARV refills, direct delivery of ART to communities, and integrating viral load testing within community-based ART distribution points.^[32]^ Besides health facilities remaining open during the restrictions, these measures restored viral load testing coverage to levels higher than before the restrictions.^[28]^ Consistent with our findings, Zakumumpa et al^[33]^ reported that during the pandemic, the use of multi-month dispensing of ARVs, home-based distribution of ARVs by a network of PLHIV, reliance on community-based drug distribution points to supply ARVs among others ensured PLHIV had sufficient doses of ARVs hence uninterrupted ART adherence and improved viral load suppression. The reduction in retention during the restrictions requires cautious interpretation for reasons. The participants benefited from multi-month dispensing of ARVs and it was permissible to refill ARVs from the nearest health facility. Therefore, such participants were likely considered non-retained. Our finding is consistent with a study that report retention declined during the restrictions^[34]^ and another that attributed the reduction to clinical, structural and psychological barriers to retention.^[35]^ Therefore, HIV control programs need to safeguard retention because it is the cornerstone for achieving optimal ART adherence and viral load suppression hence reduced HIV-related illnesses and mortality.^[36]^

Our study has several strengths. We used a rigorous methodological/analytic approach to measure the unbiased effect of the exposure and the analysis met the required model diagnostics. The study sample size is relatively large and has adequate statistical power. However, there are limitations. The weighting of the groups was based on observed covariates and several of them are unmeasured due to the analysis of secondary data. However, the results are robust to unmeasured covariates. The lack of qualitative data to explain the quantitative findings is another limitation.

The data on mortality and loss to follow-up (non-retained) require cautious interpretation as mortality data are mostly underreported and classified as lost to follow-up. We did not analyze the study outcomes as time-to-event data so under/overestimation is possible although the direction of evidence might likely remain the same. We analyzed data from an urban setting so the findings might not apply to rural settings due to socio-economic differences.

## 5. Conclusions and Recommendations

Our study in the urban setting of Kampala, the capital city of Uganda showed that during the COVID-19 pandemic restrictions, viral load testing and viral load suppression improved, while retention and mortality reduced. These findings are attributable to new measures for ART delivery and the scale-up of existing ART delivery approaches by HIV control programs.

## Acknowledgments

We thank the IDI-REC for the review and approval of the study protocol. We thank the health facility leads of the respective health facilities and the Directorate of Public Health and Management of the Kampala Capital City Authority for their administrative support.

## Author contributions

**Conceptualization:** Jonathan Izudi, Agnes N. Kiragga, Barbara Castelnuovo.

**Data curation:** Jonathan Izudi, Barbara Castelnuovo.

**Formal analysis:** Jonathan Izudi, Agnes N. Kiragga.

**Funding acquisition:** Jonathan Izudi.

**Investigation:** Jonathan Izudi, Barbara Castelnuovo.

**Methodology:** Jonathan Izudi, Agnes N. Kiragga, Barbara Castelnuovo.

**Project administration:** Agnes N. Kiragga, Philip Kalyesubula.

**Software:** Jonathan Izudi, Agnes N. Kiragga.

**Supervision:** Agnes N. Kiragga, Barbara Castelnuovo.

**Validation:** Agnes N. Kiragga, Philip Kalyesubula, Stephen Okoboi, Barbara Castelnuovo.

**Visualization:** Agnes N. Kiragga, Stephen Okoboi, Barbara Castelnuovo.

**Writing – original draft:** Jonathan Izudi, Agnes N. Kiragga, Philip Kalyesubula, Stephen Okoboi, Barbara Castelnuovo.

**Writing – review & editing:** Jonathan Izudi, Agnes N. Kiragga, Philip Kalyesubula, Stephen Okoboi, Barbara Castelnuovo.

## References

[1] Republic of Uganda. Uganda’s Emergency Response to the COVID-19 Pandemic: A Case Study. Kampala, Uganda: Ministry of Health, 2020.

[2] World Health Organization. COVID-19: Operational Guidance for Maintaining Essential Health Services During an Outbreak: Interim Guidance, 25 March 2020. World Health Organization, 2020.

[3] Burt JF Ouma J Lubyayi L. Indirect effects of COVID-19 on maternal, neonatal, child, sexual and reproductive health services in Kampala, Uganda. BMJ Global Health. 2021;6:e006102. 10.1136/bmjgh-2021-006102 PMC8406460 34452941

[4] Tumwesigye N Denis O Kaakyo M. Effects of the COVID-19 Pandemic on Health Services and Mitigation Measures in Uganda. Washington: Center for Global Development. 2021.

[5] Republic of Uganda. Consolidated Guidelines for the Prevention and Treatment of HIV and AIDS in Uganda. Kampala, Uganda: Ministry of Health, 2020.

[6] UNAIDS. Global AIDS Update: Seizing the Moment: Tackling Entrenched Inequalities to End Epidemics. Geneva, Switzerland. 2020.

[7] UNAIDS. 90-90-90: *An Ambitious Treatment Target to Help End the AIDS Epidemic*. Geneva, Switzerland 2014.

[8] Dorward J Khubone T Gate K. The impact of the COVID-19 lockdown on HIV care in 65 South African primary care clinics: an interrupted time series analysis. Lancet HIV. 2021;8:e158–65. 33549166 10.1016/S2352-3018(20)30359-3 PMC8011055

[9] Bell D Hansen KS Kiragga AN. Predicting the impact of COVID-19 and the potential impact of the public health response on disease burden in uganda. Am J Trop Med Hyg. 2020;103:1191–7. 32705975 10.4269/ajtmh.20-0546 PMC7470592

[10] World Health Organization. Preventing and Managing COVID-19 Across Long-Term Care Services: Policy Brief. Geneva, Switzerland 2020.

[11] World Health Organization. COVID-19: considerations for tuberculosis (TB) care. Date last accessed 2020;12:3–11.

[12] Staffa SJ Zurakowski D. Five steps to successfully implement and evaluate propensity score matching in clinical research studies. Anesth Analg. 2018;127:1066–73. 29324498 10.1213/ANE.0000000000002787

[13] Okoli GN Sanders RD Myles P. Demystifying propensity scores. Br J Anaesth. 2014;112:13–5. 24318697 10.1093/bja/aet290 PMC3854550

[14] White H Sabarwal S. Quasi-Experimental Design and Methods: Methodological Briefs-Impact Evaluation no. 8. 2014.

[15] Republic of Uganda. Health Sector Strategic and Investment Plan: 2010/11-2014/15. Kampala, Uganda: Ministry of Health, 2010.

[16] Republic of Uganda. National Adult HIV Quality of Care Indicators. Kampala, Uganda: Ministry of Health, 2016.

[17] The President’s Emergency Plan for AIDS Relief (PEPFAR). PEPFAR MER: Number of adults and children currently receiving antiretroviral therapy (ART). 2021; https://indicatorregistry.unaids.org/indicator/txcurrnat-subnat-percentage-adults-and-children-receiving-antiretroviral-therapy. [Access date January 24, 2022].

[18] Gertler PJ Martinez S Premand P. Impact Evaluation in Practice. World Bank Publications, 2016.

[19] R Core Team. The R Project for Statistical Computing. 2018; https://www.R-project.org/. [Access date January 5, 2022].

[20] Mao H Li L Mao MH. Package “PSW”. 2018:3–13.

[21] Olmos A Govindasamy P. A practical guide for using propensity score weighting in R. Pract Assess Res Evaluation. 2015;20:13.

[22] Olmos A Govindasamy P. Propensity scores: a practical introduction using R. J Multidiscip Eval. 2015;11:68–88.

[23] Harris H Horst SJ. A brief guide to decisions at each step of the propensity score matching process. Pract Assess Res Eval. 2016;21:4.

[24] Yao XI Wang X Speicher PJ. Reporting and guidelines in propensity score analysis: a systematic review of cancer and cancer surgical studies. JNCI: J Natl Cancer Inst. 2017;109:djw323. 28376195 10.1093/jnci/djw323 PMC6059208

[25] Des Jarlais DC Lyles C Crepaz N. Improving the reporting quality of nonrandomized evaluations of behavioral and public health interventions: the TREND statement. Am J Public Health. 2004;94:361–6. 14998794 10.2105/ajph.94.3.361 PMC1448256

[26] Ali MS Groenwold RH Klungel OH. Best (but oft-forgotten) practices: propensity score methods in clinical nutrition research. Am J Clin Nutr. 2016;104:247–58. 27413128 10.3945/ajcn.115.125914

[27] Presidential Emergency Plan for AIDS Relief PEPFAR Technical Guidance in Context of COVID-19 Pandemic. USA 2020:10–20.

[28] Lecher SL Naluguza M Mwangi C. Notes from the field: impact of the COVID-19 response on scale-up of HIV viral load testing - PEPFAR-supported countries, January-June 2020. MMWR Morb Mortal Wkly Rep. 2021;70:794–5. 34043613 10.15585/mmwr.mm7021a3 PMC8158894

[29] Adrawa N Alege JB Izudi J. Alcohol consumption increases non-adherence to ART among people living with HIV enrolled to the community-based care model in rural northern Uganda. PLoS One. 2020;15:e0242801e0242801. 33232369 10.1371/journal.pone.0242801 PMC7685449

[30] Schwartz JI Muddu M Kimera I. Impact of a COVID-19 national lockdown on integrated care for hypertension and HIV. Glob Heart. 2021;16:9. 33598389 10.5334/gh.928 PMC7863843

[31] Bajunirwe F Ayebazibwe N Mulogo E. Effectiveness of a mobile antiretroviral pharmacy and HIV care intervention on the continuum of HIV care in rural Uganda. AIDS Care. 2020;32:1111–5. 32279527 10.1080/09540121.2020.1753006

[32] GardaWorld. Uganda: lockdown measures to be eased from June 2/update 7. 2020; Available at: https://www.garda.com/crisis24/news-alerts/343631/uganda-lockdown-measures-to-be-eased-from-june-2-update-7. [Access date January 5, 2022].

[33] Zakumumpa H Tumwine C Milliam K. Dispensing antiretrovirals during Covid-19 lockdown: re-discovering community-based ART delivery models in Uganda. BMC Health Serv Res. 2021;21:692. 34256756 10.1186/s12913-021-06607-w PMC8276217

[34] Oryokot B Kazibwe A Oluka AI. COVID-19 and HIV treatment interruption: a case study of the AIDS Support Organization (TASO) Mbale clinic. World J AIDS. 2021;11:199–215.

[35] Nalubega S Kyenkya J Bagaya I. COVID-19 may exacerbate the clinical, structural and psychological barriers to retention in care among women living with HIV in rural and peri-urban settings in Uganda. BMC Infect Dis. 2021;21:1–10. 34544389 10.1186/s12879-021-06684-6 PMC8451386

[36] UNAIDS. Understanding Fast-Track: Accelerating Action to End the AIDS Epidemic by 2030. Geneva: UNAIDS, 2015.

